# When parenting fails: alexithymia and attachment states of mind in mothers of female patients with eating disorders

**DOI:** 10.3389/fpsyg.2015.01145

**Published:** 2015-08-12

**Authors:** Cecilia Serena Pace, Donatella Cavanna, Valentina Guiducci, Fabiola Bizzi

**Affiliations:** Department of Educational Science, University of Genoa, Genoa, Italy

**Keywords:** parenting, eating disorder, alexithymia, attachment states of mind, mothers

## Abstract

**Introduction:** In recent years alexithymia and attachment theory have been recognized as two parallel research lines trying to improve the information on the development and maintenance of eating disorders (EDs). However, no research has analyzed these constructs among patients’ families. In this study we compared alexithymia and attachment in mothers of patients with EDs and a control group. Further, we hypothesized that mothers of daughters with EDs with insecure and unresolved states of mind will reported high levels of alexithymia. Lastly, we explored the daughters’ evaluations of maternal alexithymia.

**Methods:** 45 mothers of ED women and 48 mothers of healthy controls (*N* = 93) matched for age and socio-demographic variables were administered by the Toronto Alexithymia Scale-20 (TAS-20) (S), while two sub-groups of “ED” mothers (*n* = 20) and “non-ED” ones (*n* = 22) were assessed by the Adult Attachment Interview (AAI). Moreover, the Observer Alexithymia Scale (OAS) was administered to the daughters for evaluating maternal alexithymia.

**Results:** Regarding alexithymia, no differences were found between ED and non-ED mothers according to the TAS-20, while ED mothers showed more unresolved AAI classifications than non-ED mothers. No correlations were found between the TAS-20 and the AAI. Lastly, ED mothers were evaluated more alexithymic by their daughters with the OAS than those in the control group, and their alexithymic traits were significantly correlated with dismissing states of mind (idealization and lack of memory) in the AAIs.

**Discussion:** Our results highlighted an interesting discrepancy among mothers with ED daughters between the low level of alexithymia provided by their self-reports and the high level of alexithymia observed by their daughters, although the OAS showed severe methodological limitations. Maternal attachment states of mind characterized by the lack of resolution of past losses could be connected to a confusing and incoherent quality of parenting.

## Introduction

Eating disorders (EDs), including anorexia nervosa (AN), bulimia nervosa (BN), and binge eating disorder (BED), are types of psychopathologies associated both with alexithymia and with attachment difficulties (O’Shaughnessy and Dallos, 2009; Zachrisson and Skårderud, 2010; Laghi et al., 2012; Nowakowski et al., 2013). Although, over the years, clinical research from psychoanalytic (Granieri and Schimmenti, 2014), systemic (Selvini Palazzoli, 1963–1995; Minuchin et al., 1978), and also cognitive-interpersonal perspectives (Schmidt and Treasure, 2006) has focused on the role played by family dynamics in the development and maintaining of an ED, very few empirical studies analyzed alexithymia and attachment representations among parents of ED patients.

Alexithymia is defined as a deficit in affect regulation, specifically referring to difficulty identifying feelings and distinguishing them from bodily sensations stemming from emotional activation, difficulty in describing their feelings to others, limited imaginative processes (“poverty of imagination”), and externally oriented cognitive style related to the stimulus (Lane et al., 2000; Luminet et al., 2006). An increasing body of clinical and empirical literature has established that patients with each type of ED (AN, BN, and BED) show higher level of alexithymia than control (non-clinical) samples, even if depressive symptoms are controlled (Cochrane et al., 1993; Jimerson et al., 1994; De Panfilis et al., 2003; Speranza et al., 2005). Alexithymia of women with ED did not correlated with their body mass index (BMI; Schmidt et al., 1993), suggesting that the high level of alexithymia of anorexic patients should not be considered an effect of starving on cognitive functioning. Specifically ED patients express *difficulty in describing feelings* (Speranza et al., 2005; Kiyotaki and Yokoyama, 2006), and especially *difficulty in identifying feelings* that emerges as the most significant predictor of the treatment outcomes (De Panfilis et al., 2003; Quinton and Wagner, 2005; Speranza et al., 2005, 2007; Kiyotaki and Yokoyama, 2006). In most studies, alexithymia was measured by the Toronto Alexithymia Scale (TAS; Taylor et al., 1985), a self-report questionnaire and its 20-item short-version (TAS-20; Bagby et al., 1994a,b), which has been already validated in Italy (Bressi et al., 1996).

Although a growing body of literature has focused on individual alexithymia among ED patients, there are only scarce and conflicting studies on alexithymia in their families. In Dahlman’s (1996) first study, the mothers of anorexic patients displayed more alexithymic (as measured by the TAS-20) than control mothers, but this result has not always been completely confirmed in subsequent studies. Guttman and Laporte (2002) found that families of women with borderline personality disorder (BPD) had the highest global scores for alexithymia on the TAS-20, followed by those with daughters with restricting-type AN and then by those without clinical problems. In Espina (2003), the parents of daughters with ED (with AN-restrictive subtype, AN-bulimic subtype, and BN) showed higher scores on the TAS-20 and its factors than the parents of control women. However, in a recent study, Balottin et al. (2014) analyzed alexithymia in anorexic adolescent patients and their parents by administering two measures: the TAS-20 and the Toronto Structured Interview for Alexithymia (TSIA; Caretti et al., 2011). Using latent-trait Rasch analysis, Balottin et al. (2014) found significant discord between the two measures: the clinical interview allowed detection of a greater level of alexithymia compared with the self-report. Indeed, in their adult parent sample in particular, no family had high alexithymic scores; as much as 75% of the families scored very low, based on the answers provided by the subjects in the TAS-20.

Recently a new questionnaire has been developed in which participants’ alexithymia is evaluated by their significant others: the Observer Alexithymia Scale (OAS; Haviland et al., 2001, 2002). The OAS was used with ED patients (Berthoz et al., 2007) showing acceptable discriminant validity and interrater reliability: patients’ OAS scores were higher than scores reported for people-in-general samples and lower than those for outpatient clinical samples. Moreover, no statistically significant OAS and TAS-20 score differences were found across diagnostic subgroups (AN restrictive type and BN), and OAS and TAS-20 total scores were moderately, positively, and significantly correlated. To our knowledge, there is no study which has measured the parental alexithymia of ED patients with the OAS.

However, the use of the OAS for assessing alexithymia is still controversial. On the one hand, both the French (Berthoz et al., 2005) and Chinese (Yao et al., 2005) translations of the OAS, administered large samples of non-clinical university students, supported that this measure appears to be a reliable and valid observer-rated alexithymia measure, and confirmed its five-factor model (distant, uninsightful, somatizing, humorless, and rigid). The authors (Berthoz et al., 2005; Yao et al., 2005) reasonably recommend that researchers collect both self- and observer-rated alexithymia data and, when possible, obtain observer reports from more than one person. On the other hand, the Dutch version of the OAS (Meganck et al., 2010), administered both to clinical and non-clinical samples, revealed adequate internal consistency and test–retest reliability, whereas inter-rater reliability, factorial validity, and concurrent validity were insufficient, inducing the authors to not recommend the OAS as an alternative alexithymia measure.

In the last 20 years, the attachment theory (Bowlby, 1973, 1988) has also received increasing attention from clinicians and researchers in the field of EDs, producing enough clinical studies so that three reviews have recently been published (O’Shaughnessy and Dallos, 2009; Zachrisson and Skårderud, 2010; Cavanna et al., 2012). Attachment researchers have specifically examined the association between attachment representations, usually defined as internal working models (IWMs), and ED diagnoses (Steele and Steele, 2008; Bakermans-Kranenburg and van IJzendoorn, 2009). Up to now, studies with ED patients have showed that their IWMs were characterized by an *over-representation of insecurity*, ranging from 70 to over 90% (Ward et al., 2001; Barone and Guiducci, 2009; Dias et al., 2011). Most studies reported high prevalence of the *dismissing* type (Cole-Detke and Kobak, 1996; Ward et al., 2001; Delogu et al., 2008; Barone and Guiducci, 2009; Delvecchio et al., 2014), characterized by a state of mind tending to minimize, derogate, and normalize attachment experiences and relationships, although other studies found both dismissing and entangled patterns (Ramacciotti et al., 2001; Zachrisson and Kulbotten, 2006). A high proportion of *entangled* subjects, characterized by a state of mind tending to emphasize attachment experiences and relationships in an angry, passive, or preoccupied manner, was specifically associated with both the purging sub-type of AN (Dias et al., 2011) and BN (Candelori and Ciocca, 1998). Furthermore, an *over-representation of unresolved loss and/or abuse* (Zachrisson and Kulbotten, 2006; Ringer and Crittenden, 2007; Barone and Guiducci, 2009; Delvecchio et al., 2014) was found, identified by local and trauma-specific disorganized speech when discussing these distressing events, such as collapse of the reasoning and discourse monitoring abilities. Most of these studies used the Adult Attachment Interview (AAI, George et al., 1985/1996) considered the “gold standard” (Bakermans-Kranenburg and van IJzendoorn, 2009) measure to assess attachment states of mind, intended as narrative IWMs of attachment.

Although attachment concepts also extend to the effectiveness in working with families, only a few studies analyzed attachment of parents with ED children. Tereno et al. (2008) found that patients’ mothers had insecure attachment styles measured by self-reports (the Adult Attachment Scale, AAS-R; Collins and Read, 1990), and, specifically, the mothers of control groups exhibited higher security than mothers of anorexic patients and lower avoidance than mothers of bulimic patients. Only two studies used the AAI with parents of ED women. Ward et al. (2001), examining the attachment status of mothers patients with severe AN using the AAI, found 83% of insecure attachment states of mind (70% dismissing) and high incidence of unresolved loss (67%) together with high levels of idealization and low levels of reflective functioning. The authors suggested that difficulty in emotional processing, exemplified by unresolved loss, idealizing defense, and poor reflective functioning, may be transmitted from mothers to daughters from childhood and act as a risk factor for the development of AN (Ward et al., 2001). These results were rather confirmed by Delogu et al. (2008) who found a prevalence of unresolved states of mind in the AAIs of mothers of anorexic adolescents and a prevalence of insecure states of mind (dismissing and entangled) among their fathers. None of these studies included control samples matched on demographic variables.

Several studies have focused on the links between alexithymia and attachment styles, measured exclusively by self-reports (Montebarocci et al., 2004; Wearden et al., 2005) in antisocial behavior (Bekker et al., 2007), alcoholic inpatients (De Rick and Vanheule, 2006), mood symptoms (Troisi et al., 2001, but only two studies examined patients with ED; Sorrentino et al., 2004; Keating et al., 2013). Sorrentino et al. (2004) found significant correlations between insecure attachment style, measured by the Attachment Style Questionnaire (ASQ, Feeney et al., 1994), alexithymia (by the TAS-20) and symptomatology in 56 ED patients, suggesting that alexithymia might be a mediating factor between insecure attachment style and psychopathology. A recent study (Keating et al., 2013) testing a model in which alexithymia mediates the relationship between attachment insecurity and body esteem and measuring by self-reported questionnaires among 300 women with ED, showed that attachment avoidance had an indirect, negative relationship to body esteem through alexithymia.

However, although in recent years alexithymia and attachment theory have been two parallel research lines that have tried to improve the information on the development and maintenance of EDs, up to now, no research has analyzed these concepts among patients’ families by investigating adult attachment representations with the AAI and testing whether ED daughters would consider their parents alexithymic, which could improve our knowledge about the functioning of this specific group of parents.

This study aimed at comparing mothers of daughters with ED diagnoses to a control group of mothers drawn from general population and matched on age, daughter’s age, socio-economic status (SES), and educational level. First, we hypothesized that the mothers of ED patients would show both higher levels of alexithymia and higher rates of insecure or unresolved attachment states of mind than controls. Second, we hypothesized that mothers of clinical group with high level of insecure and unresolved states of mind would show high levels of alexithymia. Lastly, only at explorative level, we investigated whether the ED daughters would tend to assess their mothers as more alexithymic than controls, and whether maternal alexithymia—measured by the daughters’ reports—would correlate with maternal attachment states of mind.

## Materials and Methods

### Participants

Overall, 93 participants were involved: 45 mothers of female patients primarily diagnosed with an ED (23 with AN, 22 with BN) and 48 mothers of women without clinical symptoms, who had similar socio-demographic characteristics. The study included only female participants because women have great propensity to develop an ED. Given the few studies on parents of ED patients, we decided to focus only on mothers.

Mothers of ED patients were recruited through an Eating Disorder Center (EDC) of the 3rd Health District in Genoa (northern Italy) at the first contact with the EDC’s personnel. The inclusion criteria for selecting the sample were: daughters with a primary diagnosis of ED (AN and BN) as outlined in the 4th edition of the *Diagnostic and Statistical Manual of Mental Disorders* (DSM-IV, American Psychiatric Association, 2000), aged between 16 and 25 years, and parents’ consent to participate in the study. The exclusion criteria were the following: another major diagnosis (e.g., psychotic disorders, mental retardation), onset of eating problems longer than 24 months, previously or currently receiving psychotherapeutic or medical treatment, or requiring hospital income.

Families of the control group were voluntarily recruited from the general population through public advertisement in high schools and colleges and came from a similar socio-cultural background of family of ED women. The inclusion criteria for the control group were the following: daughters’ age similar to that of the patients in the ED group, daughters should have never received psychotherapeutic or medical treatment for psychiatric symptoms, and no member of the nuclear family with a current or past history of severe physical or mental disorder.

The mothers of ED women were from 44 to 54 years old (*M* = 49, SD = 4.67). A total of 29.2% of them had attended secondary school, 45.8% graduated high school, and 25% obtained at least college degree. A majority, 71% of mothers, belonged to intact families and they were married and living with their daughter’s fathers. Their educational level and SES were coded using the Four-Factor Index of Social Position (*M* = 45, SD = 28.44, Hollingshead, 1975). No differences were found between ED’s and non-ED’s mothers with respect to the following socio-demographic variables: daughter’s age, maternal age, educational level, SES and family structure (all *p* values ranged from 0.36 to 0.88). All the participants were Caucasian, born and living in the northwest of Italy and belonged to the middle class.

Of the 93 families who participated in the alexithymia measurement, only two subgroups of the clinical group and control mothers (respectively *n* = 20 and *n* = 22) agreed to participate in the AAI. Reasons for not participating were lack of interest, long traveling distance, difficulties with being audiorecorded, time constraints, and health problems in the family. Respondents who dropped out did not differ on background and study variables, which confirmed the absence of selective attrition with respect to SES, educational level, family structure, and alexithymia scores (*p* values ranged from 0.18 to 0.75).

### Measures

#### Socio-demographic Data

Participants answered socio-demographic questions to divulge the following personal and family data: date of birth (participants and daughters), family structure, educational level, type of work, presence of psychiatric diagnosis, and psychotherapeutic or medical treatment received by nuclear family members.

#### Clinical Status of Daughters

For the clinical group, the ED diagnoses for daughters of the participants were confirmed by the Structural Clinical Interview for DSM-IV Axis I Disorders—(SCID-I; First et al., 1994), Italian version (Mazzi et al., 2000), which is a semi-structured diagnostic interview for the assessment of the primary DSM-IV Axis I disorders. It is divided into the following six self-contained modules: mood episodes, psychotic symptoms, psychotic disorders, mood disorders, substance use disorders, and anxiety, adjustment, and other disorders (First et al., 1994). One recent reliability study of the SCID-I (Lobbestael et al., 2010) reported reliability for categorical constructs, such as the DSM-IV diagnoses being assessed by the SCID, ranging from *k* = 0.60 to 0.83, according to the type of disorders.

#### Maternal Alexithymia

Alexithymia was measured through the Toronto Alexithymia Scale—20 items (TAS-20; Bagby et al., 1994a; Bressi et al., 1996), a self-report scale using a 5-point Likert scale according to which the subject provides an answer on how much he or she agrees with each item (1 = *strongly disagree*; 2 = *mildly disagree*; 3 = *neither agree nor disagree*; 4 = *mildly agree*; 5 = *strongly agree*). There are five items that are negatively keyed (items 4, 5, 10, 18, and 19). Examples of items are “I am often confused about what emotion I am feeling,” “I am able to describe my feelings easily (reversed),” “I prefer talking to people about daily activities rather than their feelings.”

The total alexithymia score is the sum of responses to all 20 items, while the score for each subscale factor is the sum of the responses to that subscale. The three subscales of the TAS-20 are (1) *difficulty identifying feeling* (7 items–1, 3, 6, 11, 9, 13, 14), (2) *difficulty describing feelings* (5 items–2, 4, 7, 12, 17), (3) *externally-oriented thinking* (8 items–5, 8, 10, 15, 16, 18, 19, 20). The TAS-20 provided cut-off scoring: participants scoring equal to or less than 51 are rated non-alexithymic, participants scoring between 52 and 60 are considered as borderline (possible alexithymia), and those with scores equal to or greater than 61 are alexithymic.

Research using the TAS-20 has demonstrated its good internal consistency (Cronbach’s alpha = 0.81), test–retest reliability (0.77, *p* < 0.01) and adequate levels of convergent and concurrent validity. The three factor structure was found to be theoretically congruent with the alexithymia construct, and the stability and replicability of the three factors have been demonstrated across clinical and non-clinical populations using confirmatory factor analysis (Parker et al., 1993; Bagby et al., 1994a).

#### Maternal Attachment States of Mind

The attachment states of mind were assessed by the AAI (George et al., 1985/1996), an hour-long, semi-structured inter-view composed of 20 questions. The interviewers inquired about participants’ relationships with their attachment figures during childhood and early attachment experiences, such as illness, upset, separation, loss, etc, asking to provide specific episodes to support their general memories. They also asked participants to reflect both on how attachment experiences had influenced their adult personality and the reasons for parents’ behavior toward them during childhood.

The AAIs were transcribed verbatim and coded on the corollary Adult Attachment Scoring and Classification System designed by Main et al. (2002) by certified and expert coders. The AAI coding system employs 17 ordinal scales of 1-9 points each, organized into two groups: (1) *subject’s inferred childhood experience* (loving, rejecting, neglecting, role reversal and pressure to achieve, each one related both to the mother and father) and (2) *current attachment states of mind* both related to the parents (idealization, anger, and derogation) and global (coherence of transcript, coherence of mind, lack of memory, metacognition, passivity, fear of loss, unresolved loss, and unresolved abuse). Therefore five *attachment classifications* are obtained: three organized -free/autonomous (F/A), dismissing (Ds), and entangled (E)- and two not organized -unresolved with respect to loss/abuse (U) and cannot classify (CC)- that could be added to the three main ones. These latter were often considered together in an unorganized (U/CC) group (Bakermans-Kranenburg and van IJzendoorn, 2009).

With regard to the psychometric properties of the AAI classifications, both the reliability (e.g., short-term stability, inter-rater consistency) and the discriminating validity with respect to gender, verbal intelligence, memory, cognitive complexity, social desirability, and overall social adjustment have been demonstrated (Bakermans-Kranenburg and van IJzendoorn, 1993; Crowell et al., 1996).

In our study, only a subgroup of 42 participants (20 mothers of ED patients and 22 control mothers) agreed to be interviewed thorough the AAI. All the transcripts were rated by a skilled coder, blind to the diagnosis and the clinical status of the participants. To obtain a reliability assessment, a second expert coder independently rated a random sample of 20 AAIs (48%), yielding a significant kappa coefficient (*k* = 0.80; *p* < 0.001) for four-way classifications (F/A, Ds, E, and U/CC).

#### Maternal Alexithymia Reported by Daughters

The alexithymia of the mothers was also assessed by the OAS Haviland et al. (2000), which was filled in by their daughters. Although our study shows some important methodological limits about the use of the OAS—above all, the lack of a third observer’s evaluation (e.g., husbands, etc)- we decided to include this measure because a broad description of the participants’ alexithymia can be clinically useful (Meganck et al., 2010), as suggested in research and clinical practice (Berthoz et al., 2007).

The OAS consists of 33 items (15 items negatively keyed) which takes raters approximately 15 min to complete. Item content is based on 13 alexithymia experts’ consensus definition of alexithymia (Haviland and Reise, 1996). The original English version of the OAS was translated into Italian by means of a translation and back-translation procedure following the guidelines of the International Test Commission (Hambleton, 1994). The OAS was translated into Italian by three English translators, native Italian speakers, who were also researchers familiar with the alexithymia concept (independent translations), the result being a negotiated Italian translation. The OAS-I was back-translated, and modifications were made in consultation with the OAS’s primary author (Haviland et al., 2000). The final version was approved by Haviland, the three original translators and a native English speaker fluent in Italian.

Each item is rated on a 4-point Likert scale from 0 (never, not at all like the person) to 3 (all of the time, completely like the person). Examples of items are “She is good at managing interpersonal relationships (reversed),” “She often speaks of physical pain or discomfort,” “She has difficulty finding the right words to describe her feelings.” The OAS global scores can range from 0 to 99, with high scores indicating greater alexithymia than low scores. The OAS consists of five subscales: distant, uninsightful, somatizing, humorless, and rigid.

Despite the above-mentioned methodological issues, the OAS scores demonstrated adequate internal consistency with Cronbach’s alpha coefficient from α = 0.84 (Haviland et al., 2000) to α = 0.88, (Yao et al., 2005), good stability (test–retest reliability) with a 2-week interval coefficient from 0.87 (Haviland et al., 2001) to 0.90 (Yao et al., 2005), acceptable interrater reliability with an intra-class correlation coefficient from 0.68 (Berthoz et al., 2007) to 0.78 (Yao et al., 2005).

### Procedure

The psychiatrist and psychologists of the EDC were informed by the research team about the selection criteria and they contacted us every time suitable families were interested to be involved in the study. The SCID-I (First et al., 1994) was administered to the outpatients to confirm their ED diagnosis. The mothers of patients who participated in the study attended two sessions at the EDC: After the first, they filled out several questionnaire, including the TAS-20 (Bagby et al., 1994a; Bressi et al., 1996), and their daughters completed the OAS (Haviland et al., 2000). At the second one, the AAI was administered only to the mothers who attended the whole procedure.

The control group had been informed—through the initial advertising—that our research team needed families with daughters between 16 and 25 years old without physical or psychological pathology to participate as a control group in a clinical study. If they wanted to participate and they passed the selection criteria, we proceeded to evaluate the maternal study variables as we did with the clinical group. This study was part of a larger research project investigating family and individual characteristics in different groups of ED patients.

At the end of the assessment, we offered a report containing a synthesis of the outcome from each instrument (TAS-20, OAS, and AAI) to the participants who completed the whole procedure. Written informed consent was obtained from all participants. The study was previously approved by the Regional Ethics Committee.

### Statistical Analysis

The results were analyzed using the Statistical Package for the Social Science (SPSS, Version 21.0; IBM Corp., Armonk, NY, USA). Some data analysis was carried out by categorizing the AAI classifications in one of the following two-way systems: secure (F/A) vs. insecure (Ds, E, U, and CC) and organized (F, Ds, and E) vs. unorganized (U and CC) categories and by using more powerful statistical tests. We used primarily non-parametric tests (e.g., Mann–Whitney U, Spearman’s rho, Fisher’s Exact test, etc), which are appropriate for statistically testing small samples, such as for this pilot study. The level of significance for all analyses was *p* < 0.05.

## Results

### Background Variables

The total TAS scores and the AAI classifications (both the two-way systems F/A vs. non-F/A and U/CC vs. non-U/CC) were not correlated with the following background variables: participants’ age, education level, SES, and family structure (*p* values ranging from 0.14 to 1.0). The total OAS score was not correlated with the following background variables: age and education level of daughters, SES, and family structure (*p* values ranging from 0.23 to 0.79). No differences emerged between daughters with AN and BN diagnoses with respect to their mothers’ AAI classifications and TAS and OAS scores (*p* values ranging from 0.14 to 0.83).

Alexithymia and attachment states of mind: comparison between mothers with “ED” daughters and mothers with “non-ED” daughters.

As Table 1 shows, no differences emerged, either in the total TAS-20 scores or in the three subscales of the TAS-20 among the two groups of mothers. The mothers of the daughters with EDs did not present a prevalence of alexithymia measured by the TAS compared to control mothers, and showed even less presence of borderline and over cut-off scores, although no significant differences were found (Table 1).

**TABLE 1:** **TAS-20’s scores and prevalence of alexithymia in EDand control group**.

	**ED mothers (*n*=45)**	**Non-EDmothers (*n*=48)**	***Mann Whitney U***	***p***
	***M* (SD)**	***M* (SD)**		
Total TAS	44.50 (8.10)	45.85 (12.81)	861.500	.88
Difficulty Identifying Feeling	14.70 (6.7)	14.05 (6.3)	875.500	.97
Difficulty Describing Feelings	13.54 (4.4)	13.58 (5.1)	865.000	.90
Externally-Oriented Thinking	17.26 (5.1)	18.22 (4.8)	772.000	.38
	***N* %**	***N* %**	***Chi Square (df)***	***p***
Alexithymia > 61	4 (9%)	8 (17%)		
Alexithymia borderline between 52–60	2 (4%)	9 (19%)	4.28 (2)	.12
No alexithymia < 51	39 (87%)	31 (64%)		

As mentioned above, a subgroup of 20 patient’s mothers and 22 of the control group mothers completed the AAI. The results were reported in Table 2. No participants were classified as CC and none of the controls was U.

**TABLE 2:** **AAI categories of ED mothers and controlgroup**.

	**F**	**Ds**	**E**	**U**	**Fourgroup *χ*^2^ (df = 3)**	**Three group *χ*^2^ (df = 2)**	**F vs. nonF Fisher Exact**	**Uvs. nonU Fisher Exact**
ED mothers (*N* = 20)	7 (35%)	5 (25%)	3 (15%)	5 (25%)	7.13, *p* = 0.07	2.99 *p* = 0.26, (ns)	*p* = 0.17 (ns)	*p* = 0.02
Non-ED mothers (*N* = 22)	12 (55%)	8 (36%)	2 (9%)	0 (%)				

The difference between the two groups of mothers on four-way AAI classification approached significance using Exact Chi Square. Given the small number of participants, we also compared the two groups of mothers on the two-way systems. No differences were found with respect to the F/A vs. non-F/A (Ds, E) classifications among the two groups of mothers with Fisher Exact test.

However, mothers of ED patients showed a higher prevalence of not-organized (U) vs. organized (F/A, Ds, and E) classifications than control mothers. Finally, mothers of ED patients indicated higher scores on the unresolved loss scale (Mann-Whitney U = 153.00, *p* < 0.01) and lower on the coherence of mind scale (Mann-Whitney U = 145.00, *p* < 0.05) than mothers of women without clinical symptoms.

Correlations between alexithymia and attachment in mothers with ED patients.

We did not find significant correlations between the global and subscales’ scores of the TAS-20 and AAI states-of-mind scales among mothers of daughters with ED (Spearman rho between –0.02 and 0.34, *p*-value ranging from 0.17 to 0.94).

Maternal alexithymia as reported by their daughters: comparison between “ED” and “non-ED” daughters and correlations with attachment states of mind.

As Table 3 shows, mothers of patients with EDs were more highly assessed in total alexithymia and were more distant and humorless than control group mothers, according to their daughters’ observations.

**TABLE 3:** **OAS’s scores of ED mothers and control group(daughter’s reports)**.

	**ED mothers (*n* = 45)**	**Non-ED mothers (*n* = 48)**	***Mann Whitney U***	***p***
	***M* (SD)**	***M* (SD)**		
Mother total OAS	43.80 (15.24)	35.31 (13.79)	722.000	0.01
*Distant* (mother)	13.98 (6.77)	9.85 (5.34)	644.000	0.00
*Uninsightful* (mother)	9.19 (5.24)	7.96 (5.09)	939.000	0.35
*Somatizing* (mother)	5.11 (3.37)	4.42 (3.08)	964.500	0.47
*Humorless* (mother)	6.85 (3.62)	4.84 (3.2)	720.500	0.01
*Rigid* (mother)	6.81 (3.49)	5.96 (3.04)	833.000	0.11

Instead, we found significant correlations between the global and subscales’ scores of the OAS and the AAI states-of-mind scales. Specifically, as showed in Table 4, mothers with higher maternal idealization in the AAI were assessed by their ED daughters as more uninsightful and those with higher scores both on the scales of fathers’ idealization and lack of memory were rated as more alexithymic.

**TABLE 4:** **Correlations between the AAI scales of states of mindand the OAS scores**.

**AAI states of mind**	**OAS scores**	**Spearman’s rho**
Idealization of mother	uninsightful	0.49*
Idealization of father	alexithymic	0.58*
Lack of memory	alexithymic	0.56*

*p < 0.05.

## Discussion

In this study, we first compared alexithymia and attachment states of mind in a group of mothers of patients with EDs matched to a control group of mothers for socio-demographic variables.

Our hypothesis was that mothers of patients with EDs would show higher levels of alexithymia but, contrary to our expectations, this was not confirmed. This result, on one hand, did not support findings from Espina (2003) who found that mothers of daughters with ED show higher scores in the TAS-20 and its factors than the controls. On the other hand, our result may be in line with those from Balottin et al. (2014) that highlighted very low levels of alexithymia among mothers of anorexic patients when they were measured by the TAS-20, which increased considerably when alexithymia was assessed via the TSIA clinical interview. In our study, noting the low percentage of alexithymia in the group of mothers of the clinical group (9.1%), we may argue that the social desiderability could have played a role in the self-evaluations reported by the mothers. Social desiderability could be increased by both the feelings of guilt and fear of judgment often reported by mothers of patients with EDs (Espindola and Blay, 2009) triggered by the context in which the research assessment were performed, that is, in the ED center where their daughters were in therapeutic treatment. We agree with Balottin et al. (2014) when they suggest “that a sense of deep crisis and distress for the daughter’s condition can lead parents to adopt denial and massive defensive attitudes toward self-administered questionnaires” (p. 1947). In this line, further studies could also integrate an evaluation of an alexithymia adding interview, like the TSIA, administered by trained clinicians. Otherwise, if we would consider maternal alexithymia also as a reactive state to stress (secondary alexithymia) and associated with the pathology of their daughters (Espina, 2003), low scores attributed to the mothers of ED patients in our study could be explained by the rather recent diagnosis of their daughters. Future longitudinal studies should collect data on alexithymia of mothers from the onset of their daughters’ ED across the time.

Regarding attachment, our hypothesis that mothers of ED patients would show higher rates of insecure and unresolved attachment states of mind (both categories and scales) than controls, was only partially confirmed. In line with our expectations, we found that mothers of ED daughters showed both significantly more unresolved AAI classifications and higher scores on the lack of loss resolution scale compared to control mothers. Contrary to our expectations, the percentage of insecure attachment classifications (65%) was not found to be significantly higher compared to control mothers (45%), although we found significantly lower scores on the coherence of mind scale among mothers of ED patients. Our results support findings from another Italian study that explored mothers’ AAIs of adolescent females with AN and found high rates of unresolved loss or trauma status (35%, Delogu et al., 2008). The English study on mothers with anorexic daughters by Ward et al. (2001) instead highlighted a very low level of free/autonomous classifications (only 2%) and a very high level of unresolved status (67%). Like Ward et al. (2001) reported, inclusion was limited to cases of women with AN symptoms severe enough to need hospitalization, which may have hypothetically selected a more insecure group of mothers. Furthermore, mothers with ED daughters appear to be more insecure and unresolved than those in the results from international and Italian meta-analyses of the AAI of “non-clinical” mothers (Bakermans-Kranenburg and van IJzendoorn, 2009; Cassibba et al., 2013). Therefore, our results confirm that unresolved states of mind play a relevant role, not only in the ED patients as several studies have already demonstrated (Ramacciotti et al., 2001; Zachrisson and Kulbotten, 2006; Ringer and Crittenden, 2007; Barone and Guiducci, 2009; Delvecchio et al., 2014), but also among their mothers. This finding deserves special attention given the link between mothers’ unresolved loss and both frightened and frightening parenting behaviors, which were considered by researcher and clinicians as relevant risk factors with respect to the children’s psychological adjustment in the developmental stages (Cowan et al., 1996; Schuengel et al., 1999; Jacobvitz et al., 2006; Ballen et al., 2007). Some authors suggested that maternal unresolved loss may be considered a severe difficulty in emotion regulation inside children–parent relationships (Cavanna et al., 2012). Specifically for these mothers with unresolved loss, when the attachment system is activated by their children, they become absorbed in their own internal and unelaborated fears, resulting in a confusing and unpredictable parenting style that does not allow their children to learn how to regulate attachment emotions, such as fear and anxiety (Cavanna, 2007). Furthermore, a theme of unresolved loss would be consistent with the older clinical literature, which emphasizes early separation difficulties in the etiology of EDs (Ward et al., 2001). Our study can not allow us any speculation about the links between mothers’ attachment states of mind and the onset of an ED in their daughters; however, an ever-increasing literature has pointed out that having mothers with free-autonomous and resolved states of mind exert positive influences on their children both in biological (Cassidy and Shaver, 2008) and adoptive families (Steele et al., 2003; Pace et al., 2012). We would suggest that enhancing attachment security and reducing unresolved losses in mothers with ED daughters should be considered one of the key points to address in the treatment of families with these patients, especially for those who are adolescents. The combination of low ability to organize an attachment relationship history in a coherent, balanced, and integrated narrative, and the lack of integration of past experience of mourning for mothers of ED patients needs to be addressed during therapeutic work with this clinical group of patients.

Our second hypothesis was that high levels of insecure states of mind would be correlated with high levels of alexithymia in mothers of ED daughters but, contrary to our expectations, we did not find any correlations between self-reported evaluation of alexithymia (TAS-20) and attachment states of mind (AAI). This finding needs to be further investigated in next research because it did not support previous studies with ED samples which revealed strong correlations between alexithymia and attachment measured by self-reports (Sorrentino et al., 2004; Keating et al., 2013). Moreover, the only study which used the AAI and the TAS-20 with a clinical sample with idiopathic spasmodic torticollis (Scheidt et al., 1999) showed that externally oriented thinking was positively correlated with dismissing attachment, and both externally oriented thinking and difficulty in communicating feelings were inversely correlated with secure attachment.

Finally, only as esplorative hypothesis, we investigated the patients’ evaluations of maternal alexithymia. First, we found that women with EDs evaluated their mothers as more alexithymic and particularly more distant and lacking a sense of humor than control women. Second, we found significant correlations between the ED daughters’ reports of their mother’s alexithymia (OAS) and scales associated with dismissing classifications of the maternal AAI, such as idealization and lack of memory. We would suggest wariness in interpreting our data by the OAS that cannot be considered as a reliable measure of maternal alexithymia for the following reasons. First, as Yao et al. (2005) recommend, obtaining observer reports from more than one person would have decisively provided more reliable data, while in our study it was not possible to have a second rater in addition to the daughters (e.g., husband, relative, close friend), to measure the mothers’ alexithymia. Second, we may speculate that patients with ED could be themselves highly alexithymic, as a wide literature review has highlighted (Nowakowski et al., 2013) and, therefore, by definition probably they are not able to provide a proper assessment of their mothers’ alexithymia. Third, an evaluation of the personality of the observers (ED and control daughters) is missing, as well as a self- and observer-reported evaluation for another independent clinical construct (e.g., anxiety, parental bonding, anger, etc). All these methodological limitations restricted the generalizability of our results that went indeed uncontrolled for the “observer” factor. Moreover, these limitations did not allow us to understand whether it is the daughter’s (biased) point of view that makes mothers of ED patients more alexithymic than those of control participants, as well as whether the clinically relevant problem (mother’s alexithymia) is specifically related to the alexithymia dimension or rather generally linked to the overall perception of the young patients about their mothers. However, beyond these highly relevant methodological limitations, from a clinical perspective, our finding with the OAS could indicate the perception that patients with EDs have of their own mothers as distant, namely lacking in interpersonal skills and affection expressions, humorless and globally alexithymic. Regardless from the actual level of maternal alexithymia, this result offers some clinical suggestions about the *inner parent* (uneasy with emotions, cold, and not playful) that these vulnerable young women have brought inside themselves.

Furthermore, our results seem to indicate that a mother with a dismissing state of mind, who tends to deactivate attachment-based feelings, experiences, and needs through minimizing and normalizing strategies, such as idealization and insistence on lack of recall, is seen by her daughter with ED as a parent with alexithymic traits. In particular, these mothers were perceived as unable to form insights, as tending to remain confused when faced with difficult situations, as frustrated when facing uncertainty, and as unable to explain their strong emotions or understand their own needs. This result deserves to be explored in future studies using self- and other- assessments both for alexithymia and attachment, including questionnaires, interviews and projective measures (Delvecchio et al., 2014).

## Limitations

Beyond the above-mentioned limitations about the OAS, this study presents several limits. First of all, because of the cross-sectional nature of the research design, we cannot make causal inferences about associations among alexithymia, attachment states of mind, and ED diagnoses. Future studies using longitudinal designs are needed to understand directional relationships among these factors. Second, the current study involved a small sample of mothers of ED girls who were outpatients in assessment phase at the time of the study, thus results from the current work may not be representative of the general ED population. Moreover, as a consequence of the small sample size, we did not differentiate between mothers of AN (restrictive and bulimic) and BN patients. Future research should involve a larger sample of mothers of ED patients and differentiate among the clinical subgroups (AN and BN) and include BED (Laghi et al., 2014, 2015). A third limitation is the absence of another non-ED psychiatric control group: although we have compared our results with mothers of “non-clinical women,” it could be argued that our findings relate to mothers of psychiatric patients in general rather than to those with EDs in particular. Further research should also address the central question of whether our findings about maternal alexithymia and attachment in this clinical group are general or specifically explain EDs. Finally, we included in our studies only assessments of mothers; as O’Shaughnessy and Dallos (2009) pointed out, fathers have largely been neglected from this area of research and further study is needed to understand the dyadic nature of alexithymia and attachment theory in both parents.

## Conclusion

The design of our study could be considered a pilot framework for future research employing larger samples of ED patients and analyzing alexithymia and attachment of their parents. We hope that our results can contribute to reducing the great risk of “blaming the mothers” that is sometimes implied in describing and interpreting connections among parental alexithymia, attachment, and psychopathology, as Zachrisson and Skårderud (2010) have suggested. Far from inducing a sense of guilt and making causal accusations upon mothers, we would suggest that involving them in the treatment of ED patients and addressing the daughter’s perception of their alexithymia together with maternal lack of coherence and unresolved loss could be beneficial for the entire family. This type of therapeutic intervention could represent the starting point both to build a more intense parent-daughter relationship and to facilitate the development of patient’s solid autonomy.

### Conflict of Interest Statement

The authors declare that the research was conducted in the absence of any commercial or financial relationships that could be construed as a potential conflict of interest.

## References

[B1] American Psychiatric Association. (2000). Diagnostic and Statistical Manual of Mental Disorders, 4th Edn, Text Revision Washington, DC: American Psychiatric Association.

[B2] Bagby R. M. Parker J. D. Taylor G. J. (1994a). The twenty-item Toronto Alexithymia Scale—I. Item selection and cross-validation of the factor structure. J. Psychosom. Res. 38, 23–32. 8126686 10.1016/0022-3999(94)90005-1

[B3] Bagby R. M. Parker J. D. Taylor G. J. (1994b). The twenty-item Toronto Alexithymia Scale—II. Convergent, discriminant and concurrent validity. J. Psychosom. Res. 38, 33–40. 8126688 10.1016/0022-3999(94)90006-x

[B4] Bakermans-Kranenburg M. J. van IJzendoorn M. H. (1993). A psychometric study of the adult attachment interview: reliability and discriminant validity. Dev. Psychol. 29, 870–879.

[B5] Bakermans-Kranenburg M. J. van IJzendoorn M. H. (2009). The first 10,000 adult attachment interviews: distributions of adult attachment representations in clinical and non-clinical groups. Attach. Hum. Dev. 11, 223–263. 10.1080/14616730902814762 19455453

[B6] Ballen N. Demers I. Bernier A. (2007). A differential analysis of the subtypes of unresolved states of mind in the adult attachment interview. J. Psychol. Trauma 5, 69–93. 10.1300/J189v05n04_04

[B7] Balottin L. Nacinovich R. Bomba M. Mannarini S. (2014). Alexithymia in parents and adolescent anorexic daughters: comparing the responses to TSIA and TAS-20 scales. Neuropsychiatr. Dis. Treat. 10, 1941–1951. 10.2147/NDT.S67642 25336959 PMC4200172

[B8] Barone L. Guiducci V. (2009). Mental representation of attachment in eating disorder: a pilot study using the adult attachment interview. Attach. Hum. Dev. 11, 405–417. 10.1080/14616730902814770 19603303

[B9] Bekker M. H. Bachrach N. Croon M. A. (2007). The relationships of antisocial behaviour with attachment styles, autonomy-connectedness, and alexithymia. J. Clin. Psychol. 63, 507–527. 10.1002/jclp.20363 17457847

[B10] Berthoz S. Haviland M. G. Riggs M. L. Perdereau F. Bungener C. (2005). Assessing alexithymia in French-speaking samples: psychometric properties of the observer alexithymia scale—French translation. Eur. Psychiatry 20, 497–502. 10.1016/j.eurpsy.2004.10.001 16310681

[B11] Berthoz S. Perdereau F. Godart N. Corcos M. Haviland M. (2007). Observer and self-rated alexithymia in eating disorders patients: levels and correspondence among three measures. J. Psychosom. Res. 62, 341–347. 10.1016/j.jpsychores.2006.10.008 17324685

[B12] Bowlby J. (1973). Attachment and Loss. Separation: Anxiety and Anger, Vol. 2. London: Penguin Books.

[B13] Bowlby J. (1988). A Secure Base: Clinical Applications of Attachment Theory. London: Routledge.

[B14] Bressi C. Taylor G. Parker J. Bressi S. Brambilla V. Aguglia E. (1996). Cross validation of the factor structure of the 20-item Toronto Alexithymia Scale: an Italian multicenter study. J. Psychosom. Res. 41, 551–559. 9032718 10.1016/s0022-3999(96)00228-0

[B15] Candelori C. Ciocca A. (1998). “Attachment and eating disorders,” in Psychotherapeutic Issues on Eating Disorders, eds Bria P. Ciocca A. de Risio S. (Rome: Societa Editrice Universo), 139–153.

[B16] Caretti V. Porcelli P. Solano L. Schimmenti A. Bagby R. M. Taylor G. J. (2011). Reliability and validity of the Toronto Structured Interview for alexithymia in a mixed clinical and nonclinical sample from Italy. Psychiatry Res. 187, 432–436. 10.1016/j.psychres.2011.02.015 21396720

[B17] Cassibba R. Sette G. Bakermans-Kranenburg M. J. van IJzendoorn M. H. (2013). Attachment the Italian way. Eur. Psychol. 18, 47–58. 10.1027/1016-9040/a000128

[B18] Cassidy J. Shaver P. R. (2008). Handbook of Attachment. Theory, Research, and Clinical Applications. New York, NY: Guilford Press.

[B19] Cavanna D. (2007). Presentazione e cura del volume di Solomon e George “L’attaccamento disorganizzato” [Presentation and treatment of Solomon and George’s “Disorganized attachment”] Bologna: Il Mulino, 7–14.

[B20] Cavanna D. Delogu A. M. Zavattini G. C. (2012). *Le prospettive dell’attaccamento nei disturbi del comportamento alimentare* [Attachment opportunities in eating disorders]. Psicol. Clin. Dello Sviluppo XVI, 3–35.

[B21] Cochrane C. Brewerton T. Wilson D. Hodges E. (1993). Alexithymia in eating disorders. Int. J. Eat. Disord. 14, 219–222. 10.1037/0022-006X.64.2.282 8401555

[B22] Cole-Detke H. Kobak R. (1996). Attachment processes in eating disorder and depression. J. Consult. Clin. Psychol. 64, 282–290. 10.1037/0022-006X.64.2.282 8871412

[B23] Collins N. L. Read S. J. (1990). Adult attachment, working models, and relationship quality in dating couples. J. Pers. Soc. Psychol. 58, 644–663. 14570079 10.1037//0022-3514.58.4.644

[B24] Cowan P. A. Cowan C. P. Cohn D. A. Pearson J. L. (1996). Parent’s attachment histories and children’s externalizing and internalizing behaviors: exploring family systems models of linkage. J. Consult. Clin. Psychol. 64, 53–63. 10.1037/0022-006X.64.1.53 8907084

[B25] Crowell J. A. Waters E. Treboux D. O’ Connor E. Colon-Downs C. Feider O. (1996). Discriminant validity of the adult attachment interview. Child Dev. 67, 2584–2599. 9022258

[B26] Dahlman K. (1996). Affective capacity in mothers of eating disorders patients. Diss. Abstr. Int. BPhysical Sci. Eng. 56, 5163–5164.

[B27] Delogu A. M. Tortolani D. Zavattini G. C. (2008). *La valutazione dell’attaccamento in famiglie anoressiche* [The assessment of attachment in anorexic families]. Infanzia Adolescenza 7, 98–109.

[B28] Delvecchio E. Di Riso D. Salcuni S. Lis A. George C. (2014). Anorexia and attachment: dysregulated defense and pathological mourning. Front. Psychol. 5:1218. 10.3389/fpsyg.2014.01218 25389412 PMC4211560

[B29] De Panfilis C. Rabbaglio P. Rossi C. Zita G. Maggini C. (2003). Body image disturbance, parental bonding and alexithymia in patients with eating disorders. Psychopathology 36, 239–246. 10.1159/000073449 14571053

[B30] De Rick A. Vanheule S. (2006). The relationship between perceived parenting, adult attachment style and alexithymia in alcoholic inpatients. Addict. Behav. 31, 1265–1270. 10.1016/j.addbeh.2005.08.010 16139435

[B31] Dias P. Soares I. Klein J. Cunha J. P. S. Roisman G. I. (2011). Autonomic correlates of attachment insecurity in a sample of women with eating disorders. Attach. Hum. Dev. 13, 155–167. 10.1080/14616734.2011.554005 21390908

[B32] Espina A. (2003). Alexithymia in parents of daughters with eating disorders: its relationships with psychopathological and personality variables. J. Psychosom. Res. 55, 553–560. 10.1016/S0022-3999(03)00016-13 14642987

[B33] Espindola C. R. Blay S. L. (2009). Family perception of anorexia and bulimia: a systematic review. Rev. Saude Publica 43, 1–9. 19503976 10.1590/s0034-89102009005000035

[B34] Feeney J. A. Noller P. Hanrahan M. (1994). “Assessing adult attachment,” in Attachment in Adults: Clinical and Developmental Perspectives, eds Sperling M. B. Berman W. H. (New York: Guilford), 122–158.

[B35] First M. B. Spitzer R. L. Gibbon M. Williams J. B. (1994). Structured Clinical Interview for DSM-IV Axis I Disorders (SCID I), Ver. 2.0. New York: Biometrics Research, New York State Psychiatric Institute.

[B36] George C. Kaplan N. Main M. (1985/1996). Adult Attachment Interview Protocol. Berkeley: University of California.

[B37] Granieri A. Schimmenti A. (2014). Mind–body splitting and eating disorders: a psychoanalytic perspective. Psychoanal. Psychother. 28, 52–70. 10.1080/02668734.2013.872172

[B38] Guttman H. Laporte L. (2002). Alexithymia, empathy, and psychological symptoms in a family context. Compr. Psychiatry 43, 448–455. 10.1053/comp.2002.35905 12439832

[B39] Hambleton R. K. (1994). Guidelines for adapting educational and psychological tests: a progress report. Eur. J. Psychol. Assess. 10, 229–244.

[B40] Haviland M. G. Reise S. P. (1996). A California Q-set alexithymia prototype and its relationship to ego-control and ego-resiliency. J. Psychosom. Res. 41, 597–608. 10.1016/S0022-3999(96)00223-221 9032723

[B41] Haviland M. G. Warren W. L. Riggs M. L. (2000). An observer scale to measure alexithymia. Psychosomatics 41, 385–392. 10.1176/appi.psy.41.5.385 11015624

[B42] Haviland M. G. Warren W. L. Riggs M. L. Gallagher M. (2001). Psychometric properties of the Observer Alexithymia Scale in a clinical sample. J. Pers. Assess. 77, 176–186. 10.1176/appi.psy.43.6.472 11562102

[B43] Haviland M. G. Warren W. L. Riggs M. L. Nitch S. R. (2002). Concurrent validity of two observer-rated alexithymia measures. Psychosomatics 43, 472–477. 10.1176/appi.psy.43.6.472 12444230

[B44] Hollingshead A. (1975). Four–Factor Index of Social Position. New Haven: Yale University.

[B45] Jacobvitz D. Leon K. Hazen N. (2006). Does expectant mother’s unresolved trauma predict frightened/frightening maternal behaviour? Risk and protective factors. Dev. Psychopathol. 18, 363–379. 10.1017/S0954579406060196 16600059

[B46] Jimerson D. Wolfe B. Franko D. Corvino N. Sifneos P. (1994). Alexithymia ratings in bulimia nervosa: clinical correlates. Psychosom. Med. 56, 90–93. 8008802 10.1097/00006842-199403000-00002

[B47] Keating L. Tasca G. A. Hill R. (2013). Structural relationships among attachment insecurity, alexithymia, and body esteem in women with eating disorders. Eat. Behav. 14, 366–373. 10.1016/j.eatbeh.2013.06.013 23910782

[B48] Kiyotaki Y. Yokoyama K. (2006). Relationships of eating disturbances to alexithymia: need for social approval, and gender identity among Japanese female undergraduate students. Pers. Individ. Differ. 41, 609–618. 10.1016/j.paid.2006.02.013

[B49] Laghi F. Baiocco R. Ghezzi E. Petrocchi N. Pace C. S. (2012). *La fiducia nell’attaccamento ai genitori e ai pari e i disturbi del comportamento alimentare in adolescenza* [Confidence in the attachment to parents and peers and eating disorders in adolescence]. Psicol. Clin. Sviluppo 16, 557–578. 10.1449/38839

[B50] Laghi F. Baiocco R. Liga F. Lonigro A. Baumgartner E. (2014). Binge eating and binge drinking behaviors: individual differences in adolescents’ identity styles. J. Health Psychol. 19, 333–343. 10.1177/1359105312470851 23405028

[B51] Laghi F. Pompili S. Baumgartner E. Baiocco R. (2015). The role of sensation seeking and motivations for eating in female and male adolescents who binge eat. Eat. Behav. 17, 119–124. 10.1016/j.eatbeh.2015.01.011 25687232

[B52] Lane R. Sechrest L. Riedel R. Shapiro D. Kaszniak A. (2000). Pervasive emotion recognition deficit common to alexithymia and the repressive coping style. Psychosom. Med. 62, 492–501. 10.1097/00006842-200007000-00007 10949094

[B53] Lobbestael J. Leurgans M. Arntz A. (2010). Inter-rater reliability of the Structured Clinical Interview for DSM-IV Axis I Disorders (SCID I) and Axis II Disorders (SCID II). Clin. Psychol. Psychother. 18, 75–79. 10.1002/cpp.693 20309842

[B54] Luminet O. Vermeulen N. Demaret C. Taylor G. J. Bagby R. M. (2006). Alexithymia and levels of processing: evidence for an overall deficit in remembering emotion words. J. Res. Pers. 40, 713–733. 10.1016/j.jrp.2005.09.001

[B55] Main M. Goldwyn R. Hesse E. (2002). Adult Attachment Scoring and Classification System, Ver. 7.1. Berkeley: University of California.

[B56] Mazzi F. Morosini P. de Girolamo G. Lussetti M. Guaraldi G. P. (2000). Intervista clinica strutturata per i disturbi dell’Asse I del DSM-IV, versione clinica [Structured Clinical Interview for Axis I Disorders (Dsm-Iv), Clinical Version]. Firenze: Organizzazioni Speciali.

[B57] Meganck R. Vanheule S. Desmet M. Inslegers R. (2010). The Observer Alexithymia Scale: a reliable and valid alternative for alexithymia measurement? J. Pers. Assess. 92, 175–185. 10.1080/00223890903510449 20155567

[B58] Minuchin S. Rosman B. L. Baker L. (1978). Psychosomatic Families: Anorexia Nervosa in Context. Cambridge, MA: Harvard University Press.

[B59] Montebarocci O. Codispoti M. Baldaro B. Rossi N. (2004). Adult attachment style and alexithymia. Pers. Individ. Differ. 36, 499–507. 10.1016/S0191-8869(03)00110-117

[B60] Nowakowski M. E. McFarlane T. Cassin S. (2013). Alexithymia and eating disorders: a critical review of the literature. J. Eat. Disord. 1, 21. 10.1186/2050-2974-1-21 24999402 PMC4081716

[B61] O’Shaughnessy R. Dallos R. (2009). Attachment research and eating disorders: a review of the literature. Clin. Child Psychol. Psychiatry 14, 559–574. 10.1177/1359104509339082 19759074

[B62] Pace C. S. Zavattini G. C. D’Alessio M. (2012). Continuity and discontinuity of attachment patterns: a short-term longitudinal pilot-study of late-adopted children and their adoptive mothers. Attach. Hum. Dev. 14, 45–61. 10.1080/14616734.2012.636658 22191606

[B63] Parker J. D. A. Bagby R. M. Taylor G. J. Endler N. S. Schmidt P. (1993). Factorial validity of the 20-item Toronto Alexithymia Scale. Eur. J. Pers. 7, 221–232.

[B64] Quinton S. Wagner H. (2005). Alexithymia, ambivalence over emotional expression, and eating attitudes. Pers. Individ. Differ. 38, 1163–1173. 10.1016/j.paid.2004.07.013

[B65] Ramacciotti A. Sorbello M. Pazzagli A. Vismara L. Mancone A. Pallanti S. (2001). Attachment processes in eating disorders. Eat. Weight Disord. 6, 166–170. 10.1007/BF03339766 11589419

[B66] Ringer F. Crittenden P. (2007). Eating disorders and attachment: the effects of hidden family processes on eating disorders. Eur. Eat. Disord. Rev. 15, 119–130. 10.1002/erv.761 17676680

[B67] Scheidt C. E. Waller E. Schnock C. Becker-Stoll F. Zimmermann P. Lucking C. H. (1999). Alexithymia and attachment representation in idiopathic spasmodic torticollis. J. Nerv. Ment. Dis. 187, 47–52. 9952253 10.1097/00005053-199901000-00008

[B68] Schmidt U. Jiwany A. Treasure J. (1993). A controlled study of alexithymia in eating disorders. Compr. Psychiatry 34, 54–58. 8425393 10.1016/0010-440x(93)90036-4

[B69] Schmidt U. Treasure J. (2006). Anorexia nervosa. Valued and visible: a cognitive-interpersonal maintenance model and its implications for research and practice. Br. J. Clin. Psychol. 45, 343–366. 10.1348/014466505X53902 17147101

[B70] Schuengel C. Bakermans-Kranenburg M. J. van IJzendoorn M. H. (1999). Frightening maternal behavior linking unresolved loss and disorganized infant attachment. J. Consult. Clin. Psychol. 67, 54–63. 10.1037/0022-006X.67.1.54 10028209

[B71] Selvini Palazzoli M. (1963–1995). Self-starvation: From Individual to Family Therapy in the Treatment of Anorexia Nervosa. Northvale, NJ: Jason Aronson.

[B72] Sorrentino D. Tonni A. Di Benedetto R. Mancuso F. Garramone S. Galderisi S. (2004). Attachment styles and alexithymia in eating and panic disorders. Eur. Psychiatry 19(Suppl. 1), 225S–225S.

[B73] Speranza M. Corcos M. Loas G. Stephan P. Guilbard O. Perez-Diaz F. (2005). Depressive personality dimensions and alexithymia in eating disorders. Psychiatry Res. 135, 153–163. 10.1016/j.psychres.2005.04.001 15913785

[B74] Speranza M. Loas G. Wallier J. Corcos M. (2007). Predictive value of alexithymia in patients with eating disorders: a 3-year prospective study. J. Psychosom. Res. 63, 365–371. 10.1016/j.jpsychores.2007.03.008 17905043

[B75] Steele M. Hodges J. Kaniuk J. Hillman S. Henderson K. (2003). Attachment representations and adoption: associations between maternal states of mind and emotion narratives in previously maltreated children. J. Child Psychother. 29, 187–205. 10.1080/0075417031000138442

[B76] Steele H. Steele M. (2008). Clinical Applications of the Adult Attachment Interview. New York: Guilford Press.

[B77] Taylor J. Ryan D. Bagby R. M. (1985). Toward the development of a new self-report alexithymia scale. Psychother. Psychosom. 44, 191–199. 3837277 10.1159/000287912

[B78] Tereno S. Soares I. Martins C. Celani M. Sampaio D. (2008). Attachment styles, memories of parental rearing and therapeutic bond: a study with eating disordered patients, their parents and therapists. Eur. Eat. Disord. Rev. 16, 49–58. 10.1002/erv.801 17879224

[B79] Troisi A. D’Argenio A. Peracchio F. Petti P. (2001). Insecure attachment and alexithymia in young men with mood symptoms. J. Nerv. Ment. Dis. 189, 311–316. 10.1097/00005053-200105000-00007 11379975

[B80] Ward A. Ramsay R. Turnbull S. Steele M. Steele H. Treasure J. (2001). Attachment in anorexia nervosa: a transgenerational perspective. Br. J. Med. Psychol. 74, 497–505. 10.1348/000711201161145 11780797

[B81] Wearden A. J. Lamberton N. Crook N. Walsh V. (2005). Adult attachment, alexithymia, and symptom reporting. An extension to the four category model of attachment. J. Psychosom. Res. 58, 279–288. 10.1016/j.jpsychores.2004.09.010 15865953

[B82] Yao S. Yi J. Zhu X. Haviland M. G. (2005). Reliability and factorial validity of the observer alexithymia scale—Chinese translation. Psychiatry Res. 134, 93–100. 10.1016/j.psychres.2004.08.010 15808294

[B83] Zachrisson H. Kulbotten G. (2006). Attachment in anorexia nervosa: an exploration of associations with eating disorder psychopathology and psychiatric symptoms. Eat. Weight. Disord. 11, 163–170. 10.1007/BF03327567 17272945

[B84] Zachrisson H. D. Skårderud F. (2010). Feelings of insecurity: review of attachment and eating disorders. Eur. Eat. Disord. Rev. 18, 97–106. 10.1002/erv.999 20148392

